# Genetic risk variants associated with in situ breast cancer

**DOI:** 10.1186/s13058-015-0596-x

**Published:** 2015-06-13

**Authors:** Daniele Campa, Myrto Barrdahl, Mia M. Gaudet, Amanda Black, Stephen J. Chanock, W. Ryan Diver, Susan M. Gapstur, Christopher Haiman, Susan Hankinson, Aditi Hazra, Brian Henderson, Robert N. Hoover, David J. Hunter, Amit D. Joshi, Peter Kraft, Loic Le Marchand, Sara Lindström, Walter Willett, Ruth C. Travis, Pilar Amiano, Afshan Siddiq, Dimitrios Trichopoulos, Malin Sund, Anne Tjønneland, Elisabete Weiderpass, Petra H. Peeters, Salvatore Panico, Laure Dossus, Regina G. Ziegler, Federico Canzian, Rudolf Kaaks

**Affiliations:** Division of Cancer Epidemiology, German Cancer Research Center (DKFZ), Im Neuenheimer Feld 581, 69120 Heidelberg, Germany; Epidemiology Research Program, American Cancer Society, 250 Williams Street NW, Atlanta, GA 30303 USA; Division of Cancer Epidemiology and Genetics, National Cancer Institute, 9000 Rockville Pike, Bethesda, MD 20892 USA; Core Genotyping Facility, Frederick National Laboratory for Cancer Research, 8717 Grovemont Circle, Gaithersburg, MD 20877 USA; Department of Preventive Medicine, Keck School of Medicine, University of Southern California, 1975 Zonal Avenue, Los Angeles, CA 90033 USA; Department of Epidemiology, University of Massachusetts-Amherst School of Public Health and Health Sciences, 715 North Pleasant Street, Amherst, MA 01003 USA; Cancer Research Center, Brigham and Women’s Hospital, 75 Francis Street, Boston, MA 02115 USA; Department of Epidemiology, Harvard School of Public Health, 677 Huntington Avenue, Boston, MA 02115 USA; Department of Medicine, Harvard Medical School, 641 Huntington Avenue, Boston, MA 02115 USA; Department of Medicine, Channing Division of Network Medicine, Brigham and Women’s Hospital, 75 Francis Street, Boston, MA 02115 USA; Cancer Research Center of Hawaii, University of Hawaii, 701 Ilalo Street, Honolulu, HI 96813 USA; Department of Nutrition, Harvard School of Public Health, 655 Huntington Avenue, Boston, MA 02115 USA; Cancer Epidemiology Unit, Nuffield Department of Population Health, University of Oxford, Roosevelt Drive, Oxford, OX3 7LF UK; Public Health Division of Gipuzkoa, BIODonostia Research Institute, Basque Health Department, Avenida Navarra 4, 20013 San Sebastian, Spain; CIBER of Epidemiology and Public Health (CIBERESP), Calle del Arzobispo Morcillo 2, 28029 Madrid, Spain; School of Public Health, Imperial College, Norfolk Place, London, W2 1PG UK; Bureau of Epidemiologic Research, Academy of Athens, 23 Alexandroupoleos Street, 115 27 Athens, Greece; Hellenic Health Foundation, 13 Kaisareias and Alexandroupoleos Street, 11527 Athens, Greece; Department of Surgical and Perioperative Sciences, Umeå University, 901 87 Umeå, Sweden; Danish Cancer Society Research Center, Strandboulevarden 49, 2100 Copenhagen, Denmark; Department of Community Medicine, Faculty of Health Sciences, University of Tromsø, The Arctic University of Norway, Hansine Hansens veg 18, 9037 Tromsø, Norway; Cancer Registry of Norway, Fridtjof Nansens vei 19, 0304 Oslo, Norway; Department of Medical Epidemiology and Biostatistics, Karolinska Institutet, Solnavägen 1, 171 77 Stockholm, Sweden; Department of Genetic Epidemiology, Folkhälsan Research Center, Haarmaninkatu 8, 00014 Helsinki, Finland; Department of Epidemiology, Julius Center for Health Sciences and Primary Care, University Medical Center, Universiteitsweg 100, 3584 CG Utrecht, The Netherlands; Dipartimento di Medicina Clinica e Chirurgia Federico II University, via Sergio Pansini 5, Naples, 80131 Italy; Inserm, Centre for research in Epidemiology and Population Health (CESP), U1018, Nutrition, Hormones and Women’s Health Team, 16 avenue Paul Vaillant Couturier, 94805 Villejuif, France; University Paris Sud, UMRS 1018, 16 avenue Paul Vaillant Couturier, 94805 Villejuif, France; IGR, 114 rue Edouard Vaillant, 94805 Villejuif, France; Genomic Epidemiology Group, German Cancer Research Center (DKFZ), Im Neuenheimer Feld 280, 69120 Heidelberg, Germany

## Abstract

**Introduction:**

Breast cancer in situ (BCIS) diagnoses, a precursor lesion for invasive breast cancer, comprise about 20 % of all breast cancers (BC) in countries with screening programs. Family history of BC is considered one of the strongest risk factors for BCIS.

**Methods:**

To evaluate the association of BC susceptibility loci with BCIS risk, we genotyped 39 single nucleotide polymorphisms (SNPs), associated with risk of invasive BC, in 1317 BCIS cases, 10,645 invasive BC cases, and 14,006 healthy controls in the National Cancer Institute’s Breast and Prostate Cancer Cohort Consortium (BPC3). Using unconditional logistic regression models adjusted for age and study, we estimated the association of SNPs with BCIS using two different comparison groups: healthy controls and invasive BC subjects to investigate whether BCIS and BC share a common genetic profile.

**Results:**

We found that five SNPs (*CDKN2BAS*-rs1011970, *FGFR2*-rs3750817, *FGFR2*-rs2981582, *TNRC9*-rs3803662, 5p12-rs10941679) were significantly associated with BCIS risk (*P* value adjusted for multiple comparisons <0.0016). Comparing invasive BC and BCIS, the largest difference was for *CDKN2BAS*-rs1011970, which showed a positive association with BCIS (OR = 1.24, 95 % CI: 1.11–1.38, *P* = 1.27 x 10^−4^) and no association with invasive BC (OR = 1.03, 95 % CI: 0.99–1.07, *P* = 0.06), with a *P* value for case-case comparison of 0.006. Subgroup analyses investigating associations with ductal carcinoma in situ (DCIS) found similar associations, albeit less significant (OR = 1.25, 95 % CI: 1.09–1.42, *P* = 1.07 x 10^−3^). Additional risk analyses showed significant associations with invasive disease at the 0.05 level for 28 of the alleles and the OR estimates were consistent with those reported by other studies.

**Conclusions:**

Our study adds to the knowledge that several of the known BC susceptibility loci are risk factors for both BCIS and invasive BC, with the possible exception of rs1011970, a putatively functional SNP situated in the *CDKN2BAS* gene that may be a specific BCIS susceptibility locus.

**Electronic supplementary material:**

The online version of this article (doi:10.1186/s13058-015-0596-x) contains supplementary material, which is available to authorized users.

## Introduction

Breast cancer in situ (BCIS) is a preinvasive breast cancer (BC) with the potential to transform into an invasive tumor within a time period that could vary between a few years to decades [1]. Only a subset of BCIS evolves into the invasive stage, and not all invasive cancers arise from BCIS [2–4]. Which factors influence the progression of BCIS to invasive BC is still unclear [2, 5, 6]. BCIS was rarely diagnosed before mass screening for BC, but since the introduction of screening they comprise about 20 % of all diagnosed BC [7, 8].

Ductal carcinoma in situ (DCIS) is the most common form of noninvasive BC. It is characterized by malignant epithelial cells inside the milk ducts of the breast. DCIS is known to be a different entity from lobular carcinoma in situ (LCIS), which is characterized by proliferation of malignant cells in the lobules of the breast [9] and is more frequently associated to lobular invasive BC than to ductal invasive BC. DCIS is generally considered a precursor lesion of invasive BC; however, a direct causality has not been firmly established because it is not possible to verify that the removal of DCIS decreases the risk of developing the invasive disease [3, 10].

BCIS is largely understudied and its etiology is poorly understood compared to invasive BC. Family history of BC is considered one of the strongest risk factors [11, 12], clearly stressing the importance of the genetic background. However, only a small number of studies have investigated the genetic risk factors specific for BCIS [13, 14] or DCIS [15, 16]. Genome-wide association studies (GWAS) including both invasive and BCIS cases tend to find similar associations between the two diseases but no specific loci have been identified for BCIS [17–19]. Findings from the Million Women Study indicated that 2p-rs4666451 may be differentially associated with invasive BC and BCIS [13], while Milne and colleagues identified the association of 5p12-rs10941679 with lower-grade BC as well as with DCIS, but not with high-grade BC [15].

With the aim of verifying whether susceptibility SNPs identified through GWAS on invasive BC are also relevant for BCIS, we selected 39 single nucleotide polymorphisms (SNPs) previously shown to be associated with invasive BC, and performed an association study on 1317 BCIS cases and 14,006 controls in the context of the US National Cancer Institute’s Breast and Prostate Cancer Cohort Consortium (BPC3). In addition, we compared the association in BCIS with 10,645 invasive BC cases to investigate whether the two types of disease share a common genetic profile or not.

## Methods

### Study population

The National Cancer Institute’s Breast and Prostate Cancer Cohort Consortium (BPC3) has been described extensively elsewhere [20]. Briefly, it consists of large, well-established cohorts assembled in Europe, Australia and the United States that have both DNA samples and extensive questionnaire information collected at baseline. Cases were women who had been diagnosed with BCIS or invasive BC after enrolment in one of the BPC3 cohorts. This study included 10,645 invasive BC cases, 1317 BCIS cases and 14,006 controls. Of the 1317 BCIS cases included in this study, 71 % had information on tumor histology. Out of these, 85 % had DCIS and 15 % had LCIS. Controls were healthy women selected from each cohort. Relevant institutional review boards from each cohort approved the project and informed consent was obtained from all participants. The names of all approving Institutional Review Boards can be found in the Acknowledgements section.

### SNP selection and genotyping

The SNPs included in this analysis were reported to show a statistically significant association with invasive BC risk (*P* <5 × 10^−7^) in at least one published study. For eight SNPs whose assays did not work satisfactorily we selected a surrogate in complete linkage disequilibrium (r^2^ = 1 in HapMap Caucasian in Europe (CEU)). In particular, for the following SNPs we have genotyped either the original SNP or the surrogate: rs4415084 (surrogate rs920329), rs9344191 (surrogate rs9449341), rs1250003 (surrogate rs704010), rs999737 (surrogate rs10483813), rs2284378 (surrogates rs8119937 and rs6059651), rs2180341 (surrogate rs9398840), rs311499 (surrogate rs311498,) and rs1917063 (surrogate rs9344208).

Genotyping was performed using TaqMan assays (Applied Biosystems, Foster City, CA, USA), as specified by the producer. Genotyping of the cases and controls was performed in four laboratories (the German Cancer Research Center (DKFZ), the University of Southern California, the US National Cancer Institute (NCI), and Harvard School of Public Health). Additional information on the genotyping techniques is given elsewhere [21]. Laboratory personnel were blinded to whether the subjects were cases or controls. Duplicate samples (approximately 8 %) were also included.

### Data filtering and statistical analysis

Concordance of the duplicate samples was evaluated and found to be greater than 99.99 % for each SNP. Each SNP was tested for Hardy-Weinberg equilibrium in the controls by study. We investigated the association between genetic variants and BCIS risk by fitting an unconditional logistic regression model, adjusted for age at recruitment and cohort (defined as study phase in NHS). Since there were only 19 BCIS patients in the European Prospective Investigation into Cancer (EPIC) we did not adjust the BCIS risk models for country. Instead, we performed sensitivity analyses, excluding EPIC. The genotypes were treated as nominal variables, comparing heterozygotes and minor allele homozygotes to the reference group major allele homozygotes. For the same reason, we did not adjust the risk models for ethnicity but performed sensitivity analyses excluding non-Caucasians.

To test if there were differences in the genetic susceptibility for the two diseases, we performed case-case analyses and subgroup analyses, matching distinct controls to BCIS cases and invasive cases, respectively. The matching factors were age at baseline, menopausal status at baseline and cohort. The same type of case-case analyses were carried out comparing allele distributions between invasive BC and DCIS cases. Furthermore, we investigated the specific associations of the alleles with DCIS.

The significance threshold was adjusted, taking into account the large number of tests carried out. Since some of the SNPs map to the same regions and might be in linkage disequilibrium, for each locus we calculated the effective number of independent SNPs, the number of effectively independent variables (M_eff_), using the SNP Spectral Decomposition approach (*simpleM* method) (13). The study-wise M_eff_ obtained was 31 and the adjusted threshold for significance was 0.05/(31) = 0.0016. All statistical tests were two-sided and all statistical analyses were performed with SAS software version 9.2 (SAS Institute, Inc., Cary, NC, USA).

### Bioinformatic analysis

We used several bioinformatic tools to assess possible functional relevance for the SNP-BCIS associations. RegulomeDB [22] and HaploReg v2B [23] were used to identify the regulatory potential of the region nearby the SNP. The GENe Expression VARiation database (Genevar) [24] was used to identify potential associations between the SNP and expression levels of nearby genes expression quantitative trait loci (eQTL).

## Results

In this study, we investigated the possible effect of 39 SNPs associated with invasive BC on the susceptibility of BCIS using 1317 BCIS cases and 14,006 healthy controls in the framework of BPC3. The relevant characteristics of the study population are presented in Table 1. The vast majority (69 %) of the study participants were postmenopausal and of European ancestry.

**Table 1:** Characteristics of the study subjects (BCIS and controls)

	CPS-II		EPIC		MEC		NHS		PLCO		Total	
	Controls	Cases	Controls	Cases	Controls	Cases	Controls	Cases	Controls	Cases	Controls	Cases
Number	3048	569	4745	19	1724	74	3630	489	859	166	14,006	1317
Ductal		297 (52 %)		14 (74 %)				367 (75 %)		114 (69 %)		792 (62 %)
Lobular		42 (8 %)		2 (10 %)				82 (17 %)		15 (9 %)		141 (11 %)
Unknown/other		230 (40 %)		3 (16 %)		74 (100 %)		40 (8 %)		37 (22 %)		384 (29 %)
White	3048	569	4745	19	574	15	3605	467	859	166	12,831	1236
Hispanic	.	.	.	.	292	10	2	.	.	.	294	10
African American	.	.	.	.	230	9	7	11	.	.	237	20
Asian	.	.	.	.	379	23	7	6	.	.	386	29
Hawaiian	.	.	.	.	249	17	.	.	.	.	249	17
Other	.	.	.	.	.	.	9	5	.	.	9	5
Age at diagnosis/recruitment, mean (sd)	61.9 (6.2)	68.81 (6.87)	54.0 (8.0)	61.16 (7.32)	57.0 (8.4)	62.86 (8.00)	57.1 (10.7)	59.04 (10.2)	62.3 (5.0)	66.13 (5.54)	57.4 (8.9)	64.41 (9.31)
ER positive	.	151	.	4	.	10	.	175	.	32	.	372
ER negative	.	22	.	.	.	2	.	35	.	9	.	68
ER not classified	.	396	.	15	.	58	.	26	.	.	.	495
ER not classified	.	.	.	.	.	4	.	253	.	125	.	382
BMI (kg/m^2^), mean (sd)	25.60 (4.93)	25.50 (4.82)	25.44 (4.31)	23.47 (3.57)	26.85 (6.16)	27.54 (5.68)	25.85 (5.20)	25.61 (5.12)	27.08 (5.38)	27.76 (5.47)	25.90 (5.05)	25.91 (5.12)
Height (m), mean (sd)	1.64 (0.063)	1.64 (0.065)	1.62 (0.066)	1.61 (0.054)	1.61 (0.070)	1.59 (0.069)	1.64 (0.061)	1.64 (0.064)	1.63 (0.063)	1.63 (0.067)	1.63 (0.066)	1.64 (0.066)
Premenopausal	108	34	1134	3	357	14	1046	172	.	.	2645	223
Postmenopausal	2902	527	2883	13	1307	56	2473	305	852	165	10,417	1066
Perimenopausal	38	8	728	3	60	4	111	12	7	1	944	28

*CPS-II* Cancer Prevention Study II, *EPIC* European Prospective Investigation into Cancer, *MEC* Multiethnic Cohort, *NHS* Nurses’ Health Study, *PLCO* Prostate, Lung, Colorectal, and Ovarian Cancer Screening Trial, *sd* standard deviation, *ER* estrogen receptor, *BMI* body mass index

We removed subjects from the NHS cohort for the analysis of *ZMIZ1*-rs1045485 and 11q13-rs614367 since the genotype distribution showed departure from the Hardy-Weinberg equilibrium among the controls (*P* = 8.4 × 10^−4^ and *P* = 6 × 10^−4^, respectively) in this cohort. All other SNPs were in Hardy-Weinberg equilibrium (*P* >0.05). The results of the sensitivity analyses showed that the exclusion of EPIC and non-Caucasian subjects did not affect the results (data not shown).

### SNP associations comparing BCIS with controls

We found significant associations (at the conventional 0.05 level) between 14 SNPs and risk of BCIS, with *P* values ranging from 0.041 (*GMBE2*-rs311499) to 3.0 x 10^−6^ (*FGFR2*-rs2981582) (Table 2). When accounting for multiple testing (*P* <0.0016), five SNPs (*CDKN2BAS*-rs1011970, *FGFR2*-rs3750817, *FGFR2*-rs2981582, *TNRC9*-rs3803662, 5p12-rs10941679) showed a statistically significant association with BCIS. Another variant (*ZNF365*-rs10995190) was very close to this significance threshold (*P* = 0.0019). None of the SNPs associated exclusively with estrogen receptor negative (ER-) BC (*C19Orf62*-rs8170, *RALY*-rs2284378, *USHBP1*-rs12982178 and *TERT*-rs10069690) or with both ER- and estrogen receptor positive (ER+) (6q14-rs13437553, 6q14-rs9344191, 6q14-rs17530068 and 20q11-rs4911414) in the literature showed an association with BCIS in this study, even at the 0.05 level.

**Table 2:** Association between the selected SNPs and risk of developing breast cancer in situ

SNP	Gene	Alleles^a^		Cases			Controls			OR (95 % CI)	*P*trend	Reference
				MM	Mm	mm^b^	MM	Mm	mm^b^			
rs11249433	*NOTCH2*	T	G	412	588	228	4757	5523	1892	1.10 (1.01-1.20)	0.022993	[34, 35]
rs10931936	*CASP8*	G	T	595	479	82	5705	4499	840	0.99 (0.90-1.09)	0.876814	[36]
rs1045485	*CASP8*	G	G	629	163	15	6653	1839	133	0.94 (0.80-1.11)	0.481823	[37]
rs13387042	Intergenic	A	G	369	590	258	3452	5942	2847	0.88 (0.81-0.96)	0.004138	[18]
rs4973768	*SLC4A7*	G	T	300	617	307	3482	6000	2728	1.07 (0.98-1.17)	0.11062	[38]
rs4415084^c^	Intergenic	G	T	384	620	218	4133	5847	2217	1.11 (1.01-1.21)	0.023783	[19]
rs10941679	Intergenic	A	G	610	478	88	6626	4601	854	1.18 (1.07-1.30)	0.001069	[19]
rs10069690	*TERT*	G	T	665	467	87	6199	4136	774	1.03 (0.93-1.13)	0.573721	[39]
rs889312	*MAP3K1*	A	G	603	506	130	6113	5020	1135	1.16 (1.06-1.27)	0.001841	[17]
rs17530068	Intergenic	T	G	727	425	86	6642	4137	648	1.01 (0.91-1.11)	0.879429	[35]
rs13437553	Intergenic	T	G	340	181	41	4628	2761	414	1.00 (0.86-1.15)	0.953341	[35]
rs1917063^d^	Intergenic	G	T	741	424	74	6933	3949	571	1.03 (0.94-1.14)	0.502161	[35]
rs9344191^e^	Intergenic	T	G	680	447	100	6365	4280	735	1.04 (0.95-1.15)	0.40587	[35]
rs2180341^f^	*RNF146*	A	G	685	458	81	6395	4084	650	1.06 (0.96-1.17)	0.250858	[40]
rs3757318	Intergenic	G	A	1019	197	8	9641	1631	54	1.19 (1.02-1.39)	0.02862	[26]
rs9383938	Intergenic	G	T	1013	212	12	9530	1820	85	1.13 (0.97-1.30)	0.108581	[35, 41]
rs2046210	Intergenic	G	T	501	565	163	5216	5494	1535	1.09 (0.99-1.19)	0.071176	[42, 43]
rs13281615	Intergenic	A	G	419	582	210	4068	5818	2232	1.00 (0.92-1.10)	0.915006	[38]
rs1562430	Intergenic	T	G	419	595	222	3821	5594	2023	1.00 (0.92-1.09)	0.992865	[26]
rs1011970	*CDKN2BAS*	G	T	793	396	42	7977	3099	319	1.24 (1.11-1.38)	0.000127	[44]
rs865686	Intergenic	T	G	481	599	157	4511	5257	1673	0.96 (0.88-1.04)	0.328473	[44]
rs2380205	Intergenic	G	T	402	597	239	3502	5637	2272	0.98 (0.90-1.06)	0.579359	[44]
rs10995190	*ZNF365*	G	A	943	277	18	8224	2923	238	0.82 (0.72-0.93)	0.001998	[44, 45]
rs16917302	*ZNF365*	A	G	1006	220	12	9313	2041	102	1.01 (0.88-1.17)	0.849328	[45, 46]
rs1250003^g^	*ZMIZ1*	A	G	444	567	227	4369	5309	1742	1.13 (1.04-1.24)	0.004096	[44, 47]
rs3750817	*FGFR2*	G	T	503	552	178	3989	5362	1804	0.86 (0.79-0.94)	0.00101	[48]
rs2981582	*FGFR2*	G	T	385	608	241	4591	5793	1847	1.23 (1.13-1.34)	0.00000283	[38]
rs3817198	*LSP1*	T	G	550	540	138	5807	5185	1178	1.03 (0.94-1.13)	0.467045	[17]
rs909116	*LSP1*	T	G	357	608	269	3125	5656	2640	0.96 (0.88-1.04)	0.309715	[26]
rs614367	Intergenic	G	T	548	188	19	5783	1909	186	1.04 (0.89-1.21)	0.63419	[49]
rs999737^h^	*RAD51L1*	G	T	751	418	58	6575	3927	656	0.89 (0.80-0.99)	0.025235	[34]
rs3803662	*TNRC9*	G	T	572	514	116	6132	4896	1070	1.20 (1.09-1.32)	0.00015	[17, 18]
rs2075555	*COL1A1*	G	A	939	265	13	8348	2582	211	0.88 (0.77-1.01)	0.062916	[50]
rs6504950	*COX11*	G	A	653	499	85	6586	4772	911	0.96 (0.88-1.06)	0.444627	[38]
rs12982178	*USHBP1*	T	G	790	391	58	7458	3649	476	1.02 (0.92-1.14)	0.667534	[35]
rs8170	*C19Orf62*	G	A	816	372	50	7699	3446	420	1.02 (0.91-1.13)	0.736771	[35]
rs2284378^i^	*RALY*	G	T	504	455	107	4955	4625	1079	0.95 (0.86-1.05)	0.298392	[35]
rs4911414	Intergenic	G	T	549	545	135	5083	5000	1295	0.95 (0.87-1.04)	0.26966	[35]
rs311499^j^	*GMEB2*	G	T	1049	169	14	9878	1491	68	1.17 (1.00-1.37)	0.04566	

*SNP* single nucleotide polymorphism, *OR,* odds ratio, *CI* confidence interval

^a^The first allele is the major, the second is the minor allele

^b^M = Major allele; m = minor allele

^c^5p12-rs4415084 or surrogate 5p12-rs920329

^d^6q14-rs1917063 or surrogate 6q14-rs9344208

^e^6q14-rs9344191 or surrogate 6q14-rs9449341

^f^ *ECHDC1R*, *NF146*-rs2180341 or surrogate *ECHDC1R*, *NF146*-rs9398840

^g^ *ZMIZ1*-rs1250003 or surrogate *ZMIZ1*-rs704010

^h^ *RAD51L1*-rs999737 or surrogate *RAD51L1-*rs10483813

^i^ *RALY*-rs2284378 or surrogate *RALY*-rs6059651, *RALY*-rs8119937

^j^ *GMEB2*-rs311499 or surrogate *GMEB2-*rs311498

### SNP associations comparing DCIS with controls

By utilizing information on tumor histology we selected the DCIS cases and investigated the associations between the alleles and risk. Of the five SNPs significantly associated with BCIS, two (*CDKN2BAS*-rs1011970, *TNRC9*-rs3803662) showed a statistically significant association with DCIS (Table S1 in Additional file 1).

### SNP associations comparing BCIS with invasive BC

Using case-case analyses to explore possible heterogeneity of associations of the SNPs with the risk of BCIS compared to invasive BC, we found no significant differences in the distribution of the genotypes of the selected SNPs by outcome (Table 3). The strongest difference was observed for *CDKN2BAS*-rs1011970, although it was not statistically significant considering multiple testing (*P* value for case-case comparison = 0.006), suggesting a stronger association of *CDKN2BAS*-rs1011970 with BCIS than with invasive BC. We also performed a subgroup analysis (BCIS vs. invasive) using matched controls in order to more clearly observe the direction of the associations between the selected SNPs and the risk of the two diseases. These latter analyses confirmed that *CDKN2BAS*-rs1011970 had a preferential association with BCIS compared to invasive BC, however, in both cases the minor T allele was associated with increased risk (Table S2 in Additional file 2).

**Table 3:** Case-case analysis between invasive breast cancer and breast cancer in situ

SNP	Gene	Alleles^a^		Invasive breast cancer			Breast cancer in situ			OR (95 % CI)	*P*trend
				MM	Mm	mm^b^	MM	Mm	mm^b^		
rs11249433	*NOTCH2*	T	G	2569	3884	1474	412	588	228	1.03 (0.94-1.13)	4,87E-01
rs10931936	*CASP8*	G	T	4470	3697	775	595	479	82	1.06 (0.96-1.18)	2,50E-01
rs1045485	*CASP8*	G	G	4570	1293	102	629	163	15	1.09 (0.92-1.28)	3,23E-01
rs13387042	Intergenic	A	G	2432	3707	1750	369	590	258	0.95 (0.88-1.04)	2,96E-01
rs4973768	*SLC4A7*	G	T	1976	4013	1932	300	617	307	0.97 (0.89-1.06)	4,86E-01
rs4415084^c^	Intergenic	G	T	2559	3863	1437	384	620	218	0.99 (0.91-1.08)	8,66E-01
rs10941679	Intergenic	A	G	4193	3143	605	610	478	88	0.99 (0.89-1.09)	8,19E-01
rs10069690	*TERT*	G	T	4243	3076	549	665	467	87	1.01 (0.91-1.11)	9,01E-01
rs889312	*MAP3K1*	A	G	3848	3306	729	603	506	130	0.96 (0.87-1.06)	4,01E-01
rs17530068	Intergenic	T	G	5171	3453	582	727	425	86	1.05 (0.95-1.17)	3,16E-01
rs13437553	Intergenic	T	G	3582	2288	361	340	181	41	1.05 (0.90-1.22)	5,60E-01
rs1917063^d^	Intergenic	G	T	5433	3301	497	741	424	74	1.02 (0.92-1.13)	7,26E-01
rs9344191^e^	Intergenic	T	G	4972	3566	645	680	447	100	1.01 (0.92-1.12)	8,36E-01
rs2180341^f^	*RNF146*	A	G	4623	2823	479	685	458	81	0.94 (0.85-1.04)	2,35E-01
rs3757318	Intergenic	G	A	7679	1443	66	1019	197	8	1.01 (0.86-1.18)	9,46E-01
rs9383938	Intergenic	G	T	7563	1568	104	1013	212	12	1.01 (0.87-1.17)	8,87E-01
rs2046210	Intergenic	G	T	3207	3633	1069	501	565	163	1.00 (0.91-1.10)	9,69E-01
rs13281615	Intergenic	A	G	2544	3773	1455	419	582	210	1.07 (0.98-1.17)	1,46E-01
rs1562430	Intergenic	T	G	3392	4347	1496	419	595	222	0.93 (0.85-1.02)	1,12E-01
rs1011970	*CDKN2BAS*	G	T	6327	2623	258	793	396	42	0.85 (0.76-0.96)	6,50E-03
rs865686	Intergenic	T	G	3847	4247	1125	481	599	157	0.93 (0.85-1.02)	1,47E-01
rs2380205	Intergenic	G	T	2961	4505	1742	402	597	239	0.99 (0.91-1.08)	8,03E-01
rs10995190	*ZNF365*	G	A	6818	2172	172	943	277	18	1.07 (0.94-1.22)	3,28E-01
rs16917302	*ZNF365*	A	G	7599	1574	86	1006	220	12	0.97 (0.84-1.13)	7,02E-01
rs1250003^g^	*ZMIZ1*	A	G	3395	4394	1432	444	567	227	0.93 (0.85-1.02)	1,20E-01
rs3750817	*FGFR2*	G	T	3146	3615	1063	503	552	178	1.01 (0.92-1.10)	8,82E-01
rs2981582	*FGFR2*	G	T	2469	3868	1546	385	608	241	1.00 (0.91-1.09)	9,66E-01
rs3817198	*LSP1*	T	G	3657	3387	821	550	540	138	0.97 (0.88-1.06)	4,67E-01
rs909116	*LSP1*	T	G	2610	4586	2040	357	608	269	1.02 (0.94-1.12)	6,31E-01
rs614367	Intergenic	G	T	5119	1937	226	548	188	19	1.14 (0.98-1.33)	9,15E-02
rs999737^h^	*RAD51L1*	G	T	4829	2702	401	751	418	58	1.04 (0.93-1.15)	5,22E-01
rs3803662	*TNRC9*	G	T	3655	3328	797	572	514	116	1.02 (0.92-1.12)	7,25E-01
rs2075555	*COL1A1*	G	A	5851	1856	165	939	265	13	1.18 (1.03-1.35)	1,41E-02
rs6504950	*COX11*	G	A	4296	3104	547	653	499	85	0.97 (0.88-1.07)	5,34E-01
rs12982178	*USHBP1*	T	G	6028	2990	327	790	391	58	0.95 (0.86-1.06)	4,04E-01
rs8170	*C19Orf62*	G	A	6237	2816	290	816	372	50	0.96 (0.85-1.07)	4,36E-01
rs2284378^i^	*RALY*	G	T	4080	3624	899	504	455	107	1.01 (0.92-1.12)	7,95E-01
rs4911414	Intergenic	G	T	4177	3954	1048	549	545	135	1.02 (0.93-1.11)	7,34E-01
rs311499^j^	*GMEB2*	G	T	7987	1162	66	1049	169	14	0.87 (0.74-1.03)	1,03E-01

*SNP* single nucleotide polymorphism, *OR,* odds ratio, *CI* confidence interval

^a^The first allele is the major, the second is the minor allele

^b^M = Major allele; m = minor allele

^c^5p12-rs4415084 or surrogate 5p12-rs920329

^d^6q14-rs1917063 or surrogate 6q14-rs9344208

^e^6q14-rs9344191 or surrogate 6q14-rs9449341

^f^ *ECHDC1R*, *NF146*-rs2180341 or surrogate *ECHDC1R*, *NF146*-rs9398840

^g^ *ZMIZ1*-rs1250003 or surrogate *ZMIZ1*-rs704010

^h^ *RAD51L1*-rs999737 or surrogate *RAD51L1-*rs10483813

^i^ *RALY*-rs2284378 or surrogate *RALY*-rs6059651, *RALY*-rs8119937

^j^ *GMEB2*-rs311499 or surrogate *GMEB2-*rs311498

When comparing invasive BC to DCIS, we observed that *CDKN2BAS*-rs1011970 showed the most promising, albeit nonsignificant association (*P* value for DCIS vs. BC case-case comparison = 0.0206, Table S3 in Additional file 3). We also noticed a stronger association of *CDKN2BAS*-rs1011970 with DCIS compared to invasive BC in the subgroup analyses (Table S4 in Additional file 4).

Additionally we also performed an association study considering only invasive BC and we found significant associations at the conventional 0.05 for 28 loci (*P* values ranging from 0.0387 to 2.27 × 10–16) (Table S2 in Additional file 2).

### Possible functional effects

For *CDKN2BAS*-rs1011970, HaploReg showed that the G to T nucleotide change of the SNP may alter the binding site for three transcription factors: FOXO4, TFC12 and p300. The Regulome DB had no data for this SNP and Genevar showed that the T allele is associated with decreased *CDKN2BA* gene expression (*P* = 0.002).

## Discussion

With the aim of better understanding the relationship of the genetic background with BCIS, we analyzed the associations of 39 previously identified BC susceptibility SNPs with BCIS risk compared to normal controls and invasive BC cases. Our general observation, as noted by others [13, 16], is that BCIS and invasive BC seem to share the same genetic risk factors. This is also supported by the fact that for the five alleles that were significantly associated (*P* <0.0016) with BCIS risk the odds ratio (OR) for BCIS risk was on the same side of 1 as the OR for invasive disease. This was true also for all the 14 alleles that were nominally (*P* <0.05) associated with BCIS risk with the exception of *GMEB2*-rs311499. However, none of the established ER- specific BC susceptibility loci were associated with BCIS risk in our study. This is not surprising because it is likely that most of the BCIS cases in our study might be ER+ (the information on this variable is extremely sparse in BPC3) and suggests that, from a genetic point of view, ER+ and ER- tumors have different risk factors even for the first stages of carcinogenesis. However, it is difficult to draw a definitive conclusion without more complete ER status data in BPC3.

When conducting case-case analysis, we observed a difference in the association of *CDKN2BAS*-rs1011970 with invasive BC and BCIS, suggesting an association with BCIS only, although this difference was not statistically significant after adjusting for multiple comparisons (*P* = 0.006). The association between rs1011970 and BC risk (OR = 1.20) was reported by Turnbull using a large GWAS conducted in European studies and was replicated in the Breast Cancer Association Consortium (BCAC; OR = 1.09) [25, 26]. The lack of association between this SNP and risk of invasive BC in our study does not appear to be due to a lack of statistical power, since with 10,645 invasive BC cases and 14,006 controls we had more than 80 % power to detect an OR of 1.1 or greater, while the ORs reported by Turnbull for this polymorphism ranged from 1.19 to 1.45, depending on the type of statistical model used. However, the results reported by Turnbull originate from cases with a family history of invasive BC, which might explain the contradictory results. These could also arise due to differing adjustments in the statistical models, different screening programs or ways of diagnosing BCIS, or by chance. Additionally, the results from Turnbull and colleagues arise from a case-control study while ours are from a prospective cohort and it has been observed that there might be discrepancies between the two study designs [27]. We found significant associations at the conventional 0.05 level with invasive BC risk for 28 of the loci. For all of these SNPs, the directions of the associations were consistent with those reported in the literature [25, 28].

From a biological point of view the association between rs1011970 and BCIS is intriguing since the SNP lies on 9p21, in an intron of the CDKN2B antisense (*CDKN2B-AS1*) gene, whose sequence overlaps with that of *CDKN2B* and flanks *CDKN2A*. These two genes encode cyclin-dependent kinase inhibitors and are frequently mutated, deleted or hypermethylated in several cancer types, including BC [29–32].

HaploReg showed that the G to T nucleotide change of rs1011970 altered the binding ability of three important cell cycle regulators (FOXO4, TFC12 and p300), possibly altering *CDKN2B* regulation. This hypothesis is corroborated by Genevar, which showed that the T allele was associated with a decreased gene expression. These data are consistent with the observation of an increased BC risk associated with the minor allele. The *CDKN2B* gene regulates cell growth and inhibits cell cycle G1 progression. The malfunctioning of this checkpoint might be particularly important in the initiation of the tumor. *CDKN2B* has been repeatedly found to be hypermethylated – a sign that the gene has been shut down, in benign lesions of the breast and in BCIS [30, 31], indicating its involvement in the early phases of carcinogenesis. Furthermore, Worsham and colleagues found that *CDKN2B* was crucial for initiating immortalization events but less important for progression to malignancy [33]. Taken together, these results suggest an involvement of the gene in early BC carcinogenesis and are consistent with our findings that the association of the SNP with BC overall could be due to its association with development of early-stage tumors, including BCIS, through the downregulation of the *CDKN2B* gene.

A limitation of this report is the fact that since the study focuses on the 39 SNPs associated with risk of invasive BC, there may be other SNPs specific for BCIS that could not be identified with this approach.

## Conclusions

In conclusion, our findings further support that the genetic variants associated with risk of BCIS and invasive BC largely overlap, with the possible exception of rs1011970, a putatively functionally relevant SNP situated in the *CDKN2BAS* gene that may be a specific BCIS locus. The discovery of a specific locus for BCIS may improve our understanding on both invasive and noninvasive BC susceptibility. However, our results for rs1011970 do not meet the criteria of statistical significance imposed by the number of tests and therefore could still reflect a chance finding.

## Additional files

Additional file 1:
**The association between the selected SNPs and risk of developing ductal breast cancer in situ.**
Additional file 2:
**Subgroup analyses, risk of breast cancer in situ and invasive breast cancer using distinct matched controls.**
Additional file 3:
**Case-case analysis between invasive breast cancer (BC) and ductal breast cancer in situ (DCIS).**
Additional file 4:
**Subgroup analyses, risk of ductal breast cancer in situ (DCIS) and invasive breast cancer using distinct matched controls.**

## Abbreviations

BC: breast cancer
BCAC: Breast Cancer Association Consortium
BCIS: breast cancer in situ
BMI: body mass index
BPC3: National Cancer Institute’s Breast and Prostate Cancer Cohort Consortium
*C19Orf62*: chromosome 19 open reading frame 62
*CASP8*: caspase 8, apoptosis-related cysteine peptidase
*CDKN2A*: cyclin-dependent kinase inhibitor 2A
*CDKN2B*: cyclin-dependent kinase inhibitor 2B
*CDKN2BAS*: CDKN2B antisense RNA 1
CEU: Caucasian in Europe
CI: confidence interval
*COL1A1*: collagen, type I, alpha 1
*COX11*: COX11 cytochrome c oxidase copper chaperone
CPS-II: Cancer Prevention Study II
DCIS: ductal carcinoma in situ
DKFZ: German Cancer Research Center
EPIC: European Prospective Investigation into Cancer
eQTL: expression quantitative trait loci
ER-: estrogen receptor negative
ER+: estrogen receptor positive
*FGFR2*: fibroblast growth factor receptor 2
*FOXO4*: forkhead box O4
*GMEB2*: glucocorticoid modulatory element-binding protein 2
GWAS: genome-wide association studies
LCIS: lobular carcinoma in situ
*LSP1*: lymphocyte-specific protein 1
*MAP3K1*: mitogen-activated protein kinase kinase kinase 1
MEC: Multiethnic Cohort
M_eff_: number of effectively independent variables
NCI: National Cancer Institute
NHS: Nurses’ Health Study
*NOTCH2*: neurogenic locus notch homolog protein 2
OR: odds ratio
PLCO: Prostate, Lung, Colorectal, and Ovarian Cancer Screening Trial
*RAD51L1*: RAD51 homolog 2
*RALY*: RALY heterogeneous nuclear ribonucleoprotein
*RNF146*: ring finger protein 146
*SLC4A7*: solute carrier family 4, sodium bicarbonate cotransporter, member 7
SNP: single nucleotide polymorphism
*TERT*: telomerase reverse transcriptase
*TFC12*: transcription factor 12
*TNRC9*: OX high mobility group box family member 3
*USHBP1*: Usher syndrome 1C binding protein 1
*ZMIZ1*: zinc finger, MIZ-type containing 1
*ZNF365*: zinc finger protein 365
