# Supplementary material for: Genetic risk variants associated with in situ breast cancer
Source: Breast Cancer Res. 2015 Jun 13;17(1):82. doi: 10.1186/s13058-015-0596-x (PMC4487950; doi:10.1186/s13058-015-0596-x)
Supplement: Additional file 1: — The association between the selected SNPs and risk of developing ductal breast cancer in situ. [file 13058_2015_596_MOESM1_ESM.doc]

**Additional file 1.** Association between the selected SNPs and risk of developing ductal breast cancer in situ.

| **SNP** | **Gene** | **Cases** | | | **Controls** | | | **OR (95% CI)** | **Ptrend** |
| --- | --- | --- | --- | --- | --- | --- | --- | --- | --- |
|  |  | **MM** | **Mm** | **mm^b^** | **MM** | **Mm** | **mm^b^** |  |  |
| rs11249433 | NOTCH2 | 256 | 372 | 140 | 3730 | 5044 | 1764 | 1.09 (0.98 to 1.21) | 1.10E-01 |
| rs10931936 | CASP8 | 371 | 316 | 54 | 5442 | 4282 | 791 | 1.03 (0.92 to 1.16) | 6.11E-01 |
| rs1045485 | CASP8 | 321 | 80 | 11 | 5296 | 1640 | 123 | 0.94 (0.76 to 1.17) | 5.71E-01 |
| rs13387042 | Intergenic | 232 | 366 | 170 | 2791 | 5288 | 2526 | 0.90 (0.81 to 1.00) | 4.33E-02 |
| rs4973768 | SLC4A7 | 188 | 375 | 202 | 2827 | 5289 | 2471 | 1.09 (0.98 to 1.21) | 1.03E-01 |
| rs4415084^c^ | Intergenic | 232 | 392 | 147 | 3796 | 5033 | 1751 | 1.17 (1.05 to 1.30) | 3.65E-03 |
| rs10941679 | Intergenic | 372 | 304 | 55 | 5940 | 3875 | 635 | 1.21 (1.07 to 1.36) | 2.00E-03 |
| rs10069690 | TERT | 436 | 282 | 52 | 5921 | 3917 | 741 | 0.97 (0.86 to 1.09) | 5.78E-01 |
| rs889312 | MAP3K1 | 393 | 304 | 80 | 5561 | 4264 | 811 | 1.11 (0.99 to 1.25) | 6.30E-02 |
| rs17530068 | Intergenic | 472 | 263 | 50 | 6325 | 3946 | 628 | 0.96 (0.85 to 1.08) | 4.87E-01 |
| rs13437553 | Intergenic | 249 | 128 | 25 | 4309 | 2571 | 397 | 0.93 (0.79 to 1.11) | 4.26E-01 |
| rs1917063^d^ | Intergenic | 476 | 266 | 43 | 6605 | 3767 | 554 | 1.00 (0.88 to 1.13) | 9.90E-01 |
| rs9344191^e^ | Intergenic | 444 | 276 | 57 | 6063 | 4080 | 710 | 0.98 (0.87 to 1.10) | 7.10E-01 |
| rs2180341^f^ | RNF146 | 436 | 274 | 58 | 6068 | 3907 | 611 | 1.07 (0.95 to 1.20) | 2.94E-01 |
| rs3757318 | Intergenic | 639 | 131 | 6 | 9186 | 1561 | 50 | 1.26 (1.05 to 1.52) | 1.30E-02 |
| rs9383938 | Intergenic | 644 | 133 | 9 | 9084 | 1739 | 83 | 1.14 (0.95 to 1.36) | 1.48E-01 |
| rs2046210 | Intergenic | 304 | 370 | 93 | 4544 | 4813 | 1269 | 1.09 (0.97 to 1.21) | 1.33E-01 |
| rs13281615 | Intergenic | 264 | 366 | 133 | 3618 | 5034 | 1831 | 1.01 (0.91 to 1.12) | 8.94E-01 |
| rs1562430 | Intergenic | 262 | 384 | 140 | 3644 | 5330 | 1939 | 1.01 (0.91 to 1.12) | 8.60E-01 |
| rs1011970 | CDKN2BAS | 504 | 245 | 31 | 7616 | 2955 | 295 | 1.25 (1.09 to 1.42) | 1.07E-03 |
| rs865686 | Intergenic | 301 | 383 | 100 | 4310 | 5004 | 1602 | 0.97 (0.87 to 1.08) | 5.51E-01 |
| rs2380205 | Intergenic | 275 | 363 | 150 | 3335 | 5370 | 2187 | 0.92 (0.83 to 1.02) | 1.31E-01 |
| rs10995190 | ZNF365 | 592 | 185 | 11 | 7851 | 2780 | 225 | 0.86 (0.74 to 1.01) | 6.02E-02 |
| rs16917302 | ZNF365 | 639 | 141 | 5 | 8883 | 1945 | 100 | 1.00 (0.83 to 1.19) | 9.64E-01 |
| rs1250003^g^ | ZMIZ1 | 278 | 367 | 140 | 4194 | 5054 | 1645 | 1.14 (1.02 to 1.26) | 1.65E-02 |
| rs3750817 | FGFR2 | 313 | 358 | 101 | 3806 | 5096 | 1726 | 0.85 (0.76 to 0.94) | 2.65E-03 |
| rs2981582 | FGFR2 | 241 | 394 | 137 | 3889 | 5066 | 1660 | 1.18 (1.06 to 1.31) | 2.36E-03 |
| rs3817198 | LSP1 | 339 | 351 | 80 | 4806 | 4656 | 1092 | 1.02 (0.91 to 1.14) | 6.96E-01 |
| rs909116 | LSP1 | 230 | 382 | 171 | 2997 | 5393 | 2508 | 0.95 (0.86 to 1.06) | 3.79E-01 |
| rs614367 | Intergenic | 310 | 105 | 10 | 5400 | 1778 | 167 | 1.02 (0.84 to 1.24) | 8.54E-01 |
| rs999737^h^ | RAD51L1 | 483 | 245 | 41 | 6240 | 3744 | 627 | 0.86 (0.76 to 0.98) | 2.33E-02 |
| rs3803662 | TNRC9 | 357 | 324 | 76 | 5542 | 4146 | 787 | 1.24 (1.10 to 1.39) | 2.43E-04 |
| rs2075555 | COL1A1 | 580 | 177 | 9 | 7957 | 2444 | 197 | 0.95 (0.81 to 1.11) | 4.94E-01 |
| rs6504950 | COX11 | 412 | 308 | 53 | 5595 | 4216 | 825 | 0.95 (0.84 to 1.06) | 3.58E-01 |
| rs12982178 | USHBP1 | 484 | 264 | 37 | 7120 | 3478 | 459 | 1.09 (0.96 to 1.24) | 1.77E-01 |
| rs8170 | C19Orf62 | 503 | 247 | 33 | 7353 | 3281 | 404 | 1.08 (0.95 to 1.23) | 2.39E-01 |
| rs2284378^i^ | RALY | 312 | 282 | 70 | 4696 | 4408 | 1026 | 0.97 (0.86 to 1.09) | 6.04E-01 |
| rs4911414 | Intergenic | 347 | 347 | 90 | 4835 | 4778 | 1239 | 0.97 (0.87 to 1.08) | 5.93E-01 |
| rs311499^j^ | GMEB2 | 664 | 110 | 7 | 9405 | 1438 | 64 | 1.17 (0.96 to 1.42) | 1.11E-01 |

| ^a^ The first allele is the major, the second is the minor allele  ^b^ M= Major allele; m= minor allele  ^c^ 5p12-rs4415084 or surrogate 5p12-rs920329  ^d^ 6q14-rs1917063 or surrogate 6q14-rs9344208  ^e^ 6q14-rs9344191 or surrogate 6q14-rs9449341 | ^f^ *ECHDC1R, NF146*-rs2180341 or surrogate *ECHDC1R, NF146*-rs9398840  ^g^ ZMIZ1-rs1250003 or surrogate ZMIZ1-rs704010  ^h^ *RAD51L1*-rs999737 or surrogate *RAD51L1-*rs10483813  ^i^ *RALY*-rs2284378 or surrogate *RALY*-rs6059651, *RALY*-rs8119937  ^j^ *GMEB2*-rs311499 or surrogate *GMEB2-*rs311498 |
| --- | --- |
