# Supplementary material for: Genetic risk variants associated with in situ breast cancer
Source: Breast Cancer Res. 2015 Jun 13;17(1):82. doi: 10.1186/s13058-015-0596-x (PMC4487950; doi:10.1186/s13058-015-0596-x)
Supplement: Additional file 2: — Subgroup analyses, risk of breast cancer in situ and invasive breast cancer using distinct matched controls. [file 13058_2015_596_MOESM2_ESM.doc]

**Additional file 2. Sub-group analyses, risk of breast cancer in situ and invasive breast cancer** using distinct matched controls

| **SNP** | **Gene or region** | **Alleles**^a^ | | **Stratum** | **Cases** | | | **Controls** | | | **OR (95% CI)** | **Ptrend** |
| --- | --- | --- | --- | --- | --- | --- | --- | --- | --- | --- | --- | --- |
|  |  |  |  |  | **MM** | **Mm** | **mm^b^** | **MM** | **Mm** | **mm^b^** |  |  |
| rs11249433 | NOTCH2 | T | G | ALL | 2979 | 4470 | 1702 | 4757 | 5523 | 1892 | 1.17 (1.12 to 1.21) | 2.12E-14 |
|  |  |  |  | In-situ | 412 | 588 | 228 | 1723 | 1429 | 484 | 1.06 (0.96 to 1.18) | 2.49E-01 |
|  |  |  |  | invasive | 2567 | 3882 | 1474 | 3034 | 4094 | 1408 | 1.12 (1.07 to 1.17) | 8.81E-07 |
| rs10931936 | CASP8 | G | T | ALL | 5063 | 4173 | 857 | 5705 | 4499 | 840 | 1.06 (1.02 to 1.11) | 8.06E-03 |
|  |  |  |  | In-situ | 595 | 479 | 82 | 1284 | 990 | 202 | 1.00 (0.89 to 1.13) | 9.88E-01 |
|  |  |  |  | invasive | 4468 | 3694 | 775 | 4421 | 3509 | 638 | 1.07 (1.02 to 1.12) | 7.70E-03 |
| rs1045485 | CASP8 | G | G | ALL | 5198 | 1456 | 117 | 6653 | 1839 | 133 | 0.98 (0.92 to 1.05) | 6.07E-01 |
|  |  |  |  | In-situ | 629 | 163 | 15 | 2311 | 497 | 43 | 0.86 (0.71 to 1.04) | 1.11E-01 |
|  |  |  |  | invasive | 4569 | 1293 | 102 | 4342 | 1342 | 90 | 0.95 (0.88 to 1.03) | 2.43E-01 |
| rs13387042 | Intergenic | A | G | ALL | 2798 | 4296 | 2007 | 3452 | 5942 | 2847 | 0.92 (0.88 to 0.95) | 1.50E-05 |
|  |  |  |  | In-situ | 369 | 590 | 258 | 1165 | 1671 | 802 | 0.88 (0.79 to 0.97) | 1.18E-02 |
|  |  |  |  | invasive | 2429 | 3706 | 1749 | 2287 | 4271 | 2045 | 0.88 (0.84 to 0.92) | 1.75E-08 |
| rs4973768 | SLC4A7 | G | T | ALL | 2276 | 4625 | 2239 | 3482 | 6000 | 2728 | 1.10 (1.05 to 1.14) | 3.87E-06 |
|  |  |  |  | In-situ | 300 | 617 | 307 | 1202 | 1681 | 748 | 1.07 (0.96 to 1.18) | 2.19E-01 |
|  |  |  |  | invasive | 1976 | 4008 | 1932 | 2280 | 4319 | 1980 | 1.07 (1.02 to 1.11) | 6.02E-03 |
| rs4415084^c^ | Intergenic | G | T | ALL | 2941 | 4483 | 1652 | 4133 | 5847 | 2217 | 1.06 (1.02 to 1.11) | 1.85E-03 |
|  |  |  |  | In-situ | 384 | 620 | 218 | 1042 | 1763 | 821 | 1.07 (0.97 to 1.19) | 1.90E-01 |
|  |  |  |  | invasive | 2557 | 3863 | 1434 | 3091 | 4084 | 1396 | 1.11 (1.06 to 1.17) | 3.86E-06 |
| rs10941679 | Intergenic | A | G | ALL | 4801 | 3620 | 691 | 6626 | 4601 | 854 | 1.11 (1.06 to 1.16) | 3.52E-06 |
|  |  |  |  | In-situ | 610 | 478 | 88 | 1762 | 1468 | 333 | 1.17 (1.03 to 1.31) | 1.28E-02 |
|  |  |  |  | invasive | 4191 | 3142 | 603 | 4864 | 3133 | 521 | 1.16 (1.10 to 1.22) | 1.05E-08 |
| rs10069690 | TERT | G | T | ALL | 4904 | 3542 | 636 | 6199 | 4136 | 774 | 1.05 (1.00 to 1.10) | 3.78E-02 |
|  |  |  |  | In-situ | 665 | 467 | 87 | 1341 | 967 | 186 | 0.94 (0.84 to 1.06) | 2.95E-01 |
|  |  |  |  | invasive | 4239 | 3075 | 549 | 4858 | 3169 | 588 | 1.06 (1.01 to 1.12) | 2.61E-02 |
| rs889312 | MAP3K1 | A | G | ALL | 4449 | 3809 | 859 | 6113 | 5020 | 1135 | 1.08 (1.03 to 1.13) | 4.78E-04 |
|  |  |  |  | In-situ | 603 | 506 | 130 | 1605 | 1570 | 466 | 1.14 (1.02 to 1.28) | 1.84E-02 |
|  |  |  |  | invasive | 3846 | 3303 | 729 | 4508 | 3450 | 669 | 1.13 (1.07 to 1.18) | 2.37E-06 |
| rs17530068 | Intergenic | T | G | ALL | 5895 | 3876 | 668 | 6642 | 4137 | 648 | 1.07 (1.02 to 1.11) | 4.55E-03 |
|  |  |  |  | In-situ | 727 | 425 | 86 | 1488 | 880 | 151 | 1.01 (0.90 to 1.14) | 8.19E-01 |
|  |  |  |  | invasive | 5168 | 3451 | 582 | 5154 | 3257 | 497 | 1.07 (1.02 to 1.12) | 8.00E-03 |
| rs13437553 | Intergenic | T | G | ALL | 3919 | 2468 | 402 | 4628 | 2761 | 414 | 1.07 (1.01 to 1.13) | 2.25E-02 |
|  |  |  |  | In-situ | 340 | 181 | 41 | 712 | 416 | 65 | 1.00 (0.84 to 1.19) | 9.82E-01 |
|  |  |  |  | invasive | 3579 | 2287 | 361 | 3916 | 2345 | 349 | 1.07 (1.01 to 1.14) | 2.26E-02 |
| rs1917063^d^ | Intergenic | G | T | ALL | 6170 | 3724 | 571 | 6933 | 3949 | 571 | 1.06 (1.01 to 1.11) | 1.05E-02 |
|  |  |  |  | In-situ | 741 | 424 | 74 | 1558 | 861 | 131 | 1.04 (0.92 to 1.17) | 5.46E-01 |
|  |  |  |  | invasive | 5429 | 3300 | 497 | 5375 | 3088 | 440 | 1.06 (1.01 to 1.11) | 2.63E-02 |
| rs9344191^e^ | Intergenic | T | G | ALL | 5648 | 4012 | 745 | 6365 | 4280 | 735 | 1.06 (1.02 to 1.11) | 4.97E-03 |
|  |  |  |  | In-situ | 680 | 447 | 100 | 1414 | 937 | 168 | 1.04 (0.92 to 1.16) | 5.33E-01 |
|  |  |  |  | invasive | 4968 | 3565 | 645 | 4951 | 3343 | 567 | 1.07 (1.02 to 1.12) | 8.67E-03 |
| rs2180341^f^ | RNF146 | A | G | ALL | 5305 | 3280 | 559 | 6395 | 4084 | 650 | 0.99 (0.95 to 1.04) | 6.79E-01 |
|  |  |  |  | In-situ | 685 | 458 | 81 | 1489 | 881 | 140 | 1.10 (0.98 to 1.24) | 1.18E-01 |
|  |  |  |  | invasive | 4620 | 2822 | 478 | 4906 | 3203 | 510 | 0.96 (0.91 to 1.01) | 1.08E-01 |
| rs3757318 | Intergenic | G | A | ALL | 8694 | 1639 | 74 | 9641 | 1631 | 54 | 1.14 (1.06 to 1.22) | 2.14E-04 |
|  |  |  |  | In-situ | 1019 | 197 | 8 | 2203 | 327 | 6 | 1.29 (1.06 to 1.57) | 1.05E-02 |
|  |  |  |  | invasive | 7675 | 1442 | 66 | 7438 | 1304 | 48 | 1.10 (1.01 to 1.18) | 2.17E-02 |
| rs9383938 | Intergenic | G | T | ALL | 8572 | 1779 | 116 | 9530 | 1820 | 85 | 1.12 (1.05 to 1.19) | 8.69E-04 |
|  |  |  |  | In-situ | 1013 | 212 | 12 | 2145 | 391 | 13 | 1.10 (0.92 to 1.32) | 2.93E-01 |
|  |  |  |  | invasive | 7559 | 1567 | 104 | 7385 | 1429 | 72 | 1.10 (1.03 to 1.19) | 7.66E-03 |
| rs2046210 | Intergenic | G | T | ALL | 3705 | 4197 | 1231 | 5216 | 5494 | 1535 | 1.08 (1.04 to 1.12) | 2.52E-04 |
|  |  |  |  | In-situ | 501 | 565 | 163 | 1532 | 1580 | 509 | 1.05 (0.94 to 1.17) | 3.93E-01 |
|  |  |  |  | invasive | 3204 | 3632 | 1068 | 3684 | 3914 | 1026 | 1.09 (1.04 to 1.14) | 2.84E-04 |
| rs13281615 | Intergenic | A | G | ALL | 2963 | 4353 | 1663 | 4068 | 5818 | 2232 | 1.03 (0.99 to 1.08) | 9.26E-02 |
|  |  |  |  | In-situ | 419 | 582 | 210 | 1124 | 1770 | 714 | 1.03 (0.92 to 1.14) | 6.38E-01 |
|  |  |  |  | invasive | 2544 | 3771 | 1453 | 2944 | 4048 | 1518 | 1.05 (1.01 to 1.10) | 2.71E-02 |
| rs1562430 | Intergenic | T | G | ALL | 3808 | 4941 | 1717 | 3821 | 5594 | 2023 | 0.91 (0.88 to 0.95) | 4.78E-06 |
|  |  |  |  | In-situ | 419 | 595 | 222 | 843 | 1266 | 435 | 1.01 (0.91 to 1.12) | 8.25E-01 |
|  |  |  |  | invasive | 3389 | 4346 | 1495 | 2978 | 4328 | 1588 | 0.91 (0.87 to 0.95) | 9.57E-06 |
| rs1011970 | CDKN2BAS | G | T | ALL | 7118 | 3018 | 299 | 7977 | 3099 | 319 | 1.07 (1.02 to 1.13) | 5.12E-03 |
|  |  |  |  | In-situ | 793 | 396 | 42 | 1800 | 668 | 79 | 1.31 (1.14 to 1.50) | 1.03E-04 |
|  |  |  |  | invasive | 6325 | 2622 | 257 | 6177 | 2431 | 240 | 1.06 (1.00 to 1.12) | 5.67E-02 |
| rs865686 | Intergenic | T | G | ALL | 4327 | 4843 | 1281 | 4511 | 5257 | 1673 | 0.91 (0.87 to 0.95) | 2.13E-06 |
|  |  |  |  | In-situ | 481 | 599 | 157 | 976 | 1198 | 374 | 0.95 (0.86 to 1.06) | 3.70E-01 |
|  |  |  |  | invasive | 3846 | 4244 | 1124 | 3535 | 4059 | 1299 | 0.90 (0.86 to 0.94) | 1.72E-06 |
| rs2380205 | Intergenic | G | T | ALL | 3362 | 5100 | 1979 | 3502 | 5637 | 2272 | 0.95 (0.91 to 0.99) | 8.16E-03 |
|  |  |  |  | In-situ | 402 | 597 | 239 | 810 | 1256 | 473 | 1.00 (0.90 to 1.11) | 9.78E-01 |
|  |  |  |  | invasive | 2960 | 4503 | 1740 | 2692 | 4381 | 1799 | 0.93 (0.89 to 0.97) | 9.66E-04 |
| rs10995190 | ZNF365 | G | A | ALL | 7757 | 2448 | 190 | 8224 | 2923 | 238 | 0.89 (0.84 to 0.94) | 1.47E-05 |
|  |  |  |  | In-situ | 943 | 277 | 18 | 1807 | 691 | 53 | 0.79 (0.68 to 0.92) | 2.45E-03 |
|  |  |  |  | invasive | 6814 | 2171 | 172 | 6417 | 2232 | 185 | 0.90 (0.84 to 0.95) | 4.70E-04 |
| rs16917302 | ZNF365 | A | G | ALL | 8600 | 1794 | 98 | 9313 | 2041 | 102 | 0.96 (0.90 to 1.03) | 2.56E-01 |
|  |  |  |  | In-situ | 1006 | 220 | 12 | 2075 | 448 | 23 | 0.99 (0.83 to 1.18) | 9.38E-01 |
|  |  |  |  | invasive | 7594 | 1574 | 86 | 7238 | 1593 | 79 | 0.96 (0.89 to 1.03) | 2.51E-01 |
| rs1250003^g^ | ZMIZ1 | A | G | ALL | 3837 | 4959 | 1659 | 4369 | 5309 | 1742 | 1.05 (1.01 to 1.09) | 1.86E-02 |
|  |  |  |  | In-situ | 444 | 567 | 227 | 940 | 1208 | 393 | 1.08 (0.97 to 1.19) | 1.62E-01 |
|  |  |  |  | invasive | 3393 | 4392 | 1432 | 3429 | 4101 | 1349 | 1.05 (1.01 to 1.10) | 1.92E-02 |
| rs3750817 | FGFR2 | G | T | ALL | 3648 | 4164 | 1240 | 3989 | 5362 | 1804 | 0.86 (0.83 to 0.90) | 2.92E-13 |
|  |  |  |  | In-situ | 503 | 552 | 178 | 900 | 1236 | 409 | 0.85 (0.77 to 0.95) | 2.75E-03 |
|  |  |  |  | invasive | 3145 | 3612 | 1062 | 3089 | 4126 | 1395 | 0.86 (0.82 to 0.90) | 4.94E-10 |
| rs2981582 | FGFR2 | G | T | ALL | 2853 | 4473 | 1786 | 4591 | 5793 | 1847 | 1.24 (1.19 to 1.29) | 6.08E-26 |
|  |  |  |  | In-situ | 385 | 608 | 241 | 1450 | 1676 | 504 | 1.29 (1.16 to 1.43) | 2.02E-06 |
|  |  |  |  | invasive | 2468 | 3865 | 1545 | 3141 | 4117 | 1343 | 1.21 (1.16 to 1.27) | 2.27E-16 |
| rs3817198 | LSP1 | T | G | ALL | 4206 | 3924 | 958 | 5807 | 5185 | 1178 | 1.02 (0.98 to 1.07) | 2.76E-01 |
|  |  |  |  | In-situ | 550 | 540 | 138 | 1896 | 1394 | 321 | 1.02 (0.92 to 1.14) | 6.71E-01 |
|  |  |  |  | invasive | 3656 | 3384 | 820 | 3911 | 3791 | 857 | 0.99 (0.94 to 1.04) | 6.24E-01 |
| rs909116 | LSP1 | T | G | ALL | 2965 | 5192 | 2308 | 3125 | 5656 | 2640 | 0.96 (0.92 to 1.00) | 3.24E-02 |
|  |  |  |  | In-situ | 357 | 608 | 269 | 711 | 1235 | 597 | 0.92 (0.83 to 1.02) | 9.56E-02 |
|  |  |  |  | invasive | 2608 | 4584 | 2039 | 2414 | 4421 | 2043 | 0.97 (0.93 to 1.01) | 1.43E-01 |
| rs614367 | Intergenic | G | T | ALL | 5666 | 2125 | 245 | 5783 | 1909 | 186 | 1.15 (1.08 to 1.22) | 1.05E-05 |
|  |  |  |  | In-situ | 548 | 188 | 19 | 1326 | 454 | 40 | 1.07 (0.88 to 1.29) | 4.96E-01 |
|  |  |  |  | invasive | 5118 | 1937 | 226 | 4457 | 1455 | 146 | 1.15 (1.07 to 1.23) | 5.46E-05 |
| rs999737^h^ | RAD51L1 | G | T | ALL | 5576 | 3119 | 459 | 6575 | 3927 | 656 | 0.92 (0.88 to 0.97) | 7.94E-04 |
|  |  |  |  | In-situ | 751 | 418 | 58 | 1533 | 867 | 158 | 0.90 (0.80 to 1.02) | 1.08E-01 |
|  |  |  |  | invasive | 4825 | 2701 | 401 | 5042 | 3060 | 498 | 0.93 (0.88 to 0.98) | 5.73E-03 |
| rs3803662 | TNRC9 | G | T | ALL | 4224 | 3841 | 912 | 6132 | 4896 | 1070 | 1.17 (1.12 to 1.22) | 1.02E-12 |
|  |  |  |  | In-situ | 572 | 514 | 116 | 1704 | 1477 | 426 | 1.27 (1.13 to 1.42) | 4.30E-05 |
|  |  |  |  | invasive | 3652 | 3327 | 796 | 4428 | 3419 | 644 | 1.19 (1.13 to 1.25) | 4.05E-12 |
| rs2075555 | COL1A1 | G | A | ALL | 6785 | 2121 | 178 | 8348 | 2582 | 211 | 1.02 (0.96 to 1.08) | 5.28E-01 |
|  |  |  |  | In-situ | 939 | 265 | 13 | 1869 | 629 | 50 | 0.83 (0.71 to 0.97) | 1.99E-02 |
|  |  |  |  | invasive | 5846 | 1856 | 165 | 6479 | 1953 | 161 | 1.07 (1.00 to 1.14) | 3.87E-02 |
| rs6504950 | COX11 | G | A | ALL | 4946 | 3601 | 632 | 6586 | 4772 | 911 | 0.97 (0.93 to 1.01) | 1.31E-01 |
|  |  |  |  | In-situ | 653 | 499 | 85 | 2050 | 1343 | 251 | 0.99 (0.88 to 1.11) | 8.95E-01 |
|  |  |  |  | invasive | 4293 | 3102 | 547 | 4536 | 3429 | 660 | 0.94 (0.90 to 0.99) | 2.74E-02 |
| rs12982178 | USHBP1 | T | G | ALL | 6813 | 3381 | 385 | 7458 | 3649 | 476 | 0.99 (0.94 to 1.03) | 5.65E-01 |
|  |  |  |  | In-situ | 790 | 391 | 58 | 1663 | 779 | 109 | 1.04 (0.92 to 1.19) | 5.07E-01 |
|  |  |  |  | invasive | 6023 | 2990 | 327 | 5795 | 2870 | 367 | 0.97 (0.92 to 1.03) | 2.97E-01 |
| rs8170 | C19Orf62 | G | A | ALL | 7048 | 3188 | 340 | 7699 | 3446 | 420 | 0.98 (0.94 to 1.03) | 5.31E-01 |
|  |  |  |  | In-situ | 816 | 372 | 50 | 1705 | 749 | 89 | 1.04 (0.91 to 1.19) | 5.58E-01 |
|  |  |  |  | invasive | 6232 | 2816 | 290 | 5994 | 2697 | 331 | 0.97 (0.92 to 1.02) | 2.74E-01 |
| rs2284378^i^ | RALY | G | T | ALL | 4581 | 4077 | 1006 | 4955 | 4625 | 1079 | 0.99 (0.95 to 1.03) | 6.23E-01 |
|  |  |  |  | In-situ | 504 | 455 | 107 | 769 | 706 | 199 | 0.92 (0.81 to 1.03) | 1.55E-01 |
|  |  |  |  | invasive | 4077 | 3622 | 899 | 4186 | 3919 | 880 | 1.00 (0.96 to 1.05) | 9.70E-01 |
| rs4911414 | Intergenic | G | T | ALL | 4723 | 4498 | 1182 | 5083 | 5000 | 1295 | 0.99 (0.95 to 1.03) | 4.99E-01 |
|  |  |  |  | In-situ | 549 | 545 | 135 | 1082 | 1106 | 335 | 0.92 (0.83 to 1.03) | 1.39E-01 |
|  |  |  |  | invasive | 4174 | 3953 | 1047 | 4001 | 3894 | 960 | 1.00 (0.96 to 1.05) | 9.98E-01 |
| rs311499^j^ | GMEB2 | G | T | ALL | 9031 | 1331 | 80 | 9878 | 1491 | 68 | 1.00 (0.93 to 1.08) | 9.24E-01 |
|  |  |  |  | In-situ | 1049 | 169 | 14 | 2235 | 294 | 19 | 1.18 (0.97 to 1.43) | 9.60E-02 |
|  |  |  |  | invasive | 7982 | 1162 | 66 | 7643 | 1197 | 49 | 0.98 (0.90 to 1.06) | 6.34E-01 |

| ^a^ The first allele is the major, the second is the minor allele  ^b^ M= Major allele; m= minor allele  ^c^ 5p12-rs4415084 or surrogate 5p12-rs920329  ^d^ 6q14-rs1917063 or surrogate 6q14-rs9344208  ^e^ 6q14-rs9344191 or surrogate 6q14-rs9449341 | ^f^ *ECHDC1R, NF146*-rs2180341 or surrogate *ECHDC1R, NF146*-rs9398840  ^g^ ZMIZ1-rs1250003 or surrogate ZMIZ1-rs704010  ^h^ *RAD51L1*-rs999737 or surrogate *RAD51L1-*rs10483813  ^i^ *RALY*-rs2284378 or surrogate *RALY*-rs6059651, *RALY*-rs8119937  ^j^ *GMEB2*-rs311499 or surrogate *GMEB2-*rs311498 |
| --- | --- |
