# Supplementary material for: Genetic risk variants associated with in situ breast cancer
Source: Breast Cancer Res. 2015 Jun 13;17(1):82. doi: 10.1186/s13058-015-0596-x (PMC4487950; doi:10.1186/s13058-015-0596-x)
Supplement: Additional file 3: — Case-case analysis between invasive breast cancer (BC) and ductal breast cancer in situ (DCIS). [file 13058_2015_596_MOESM3_ESM.doc]

**Additional file 3. Case-case analysis between invasive breast cancer (BC) and ductal breast cancer in situ (DCIS)**.

| **SNP** | **Gene** | **Invasive BC**  **MM Mm mm^b^** | | | **DCIS**  **MM Mm mm^b^** | | | **OR (95% CI)** | **Ptrend** |
| --- | --- | --- | --- | --- | --- | --- | --- | --- | --- |
| rs11249433 | NOTCH2 | 2569 | 3884 | 1474 | 256 | 372 | 140 | 1.03 (0.92 to 1.15) | 6.14E-01 |
| rs10931936 | CASP8 | 4470 | 3697 | 775 | 371 | 316 | 54 | 1.02 (0.90 to 1.15) | 7.56E-01 |
| rs1045485 | CASP8 | 4570 | 1293 | 102 | 321 | 80 | 11 | 1.03 (0.83 to 1.28) | 7.91E-01 |
| rs13387042 | Intergenic | 2432 | 3707 | 1750 | 232 | 366 | 170 | 0.93 (0.84 to 1.03) | 1.86E-01 |
| rs4973768 | SLC4A7 | 1976 | 4013 | 1932 | 188 | 375 | 202 | 0.95 (0.85 to 1.06) | 3.29E-01 |
| rs4415084^c^ | Intergenic | 2559 | 3863 | 1437 | 232 | 392 | 147 | 0.94 (0.85 to 1.05) | 2.90E-01 |
| rs10941679 | Intergenic | 4193 | 3143 | 605 | 372 | 304 | 55 | 0.97 (0.86 to 1.09) | 5.96E-01 |
| rs10069690 | TERT | 4243 | 3076 | 549 | 436 | 282 | 52 | 1.07 (0.95 to 1.22) | 2.57E-01 |
| rs889312 | MAP3K1 | 3848 | 3306 | 729 | 393 | 304 | 80 | 1.01 (0.89 to 1.13) | 9.22E-01 |
| rs17530068 | Intergenic | 5171 | 3453 | 582 | 472 | 263 | 50 | 1.10 (0.97 to 1.25) | 1.21E-01 |
| rs13437553 | Intergenic | 3582 | 2288 | 361 | 249 | 128 | 25 | 1.12 (0.93 to 1.34) | 2.25E-01 |
| rs1917063^d^ | Intergenic | 5433 | 3301 | 497 | 476 | 266 | 43 | 1.05 (0.93 to 1.20) | 4.27E-01 |
| rs9344191^e^ | Intergenic | 4972 | 3566 | 645 | 444 | 276 | 57 | 1.07 (0.95 to 1.21) | 2.62E-01 |
| rs2180341^f^ | RNF146 | 4623 | 2823 | 479 | 436 | 274 | 58 | 0.93 (0.83 to 1.06) | 2.76E-01 |
| rs3757318 | Intergenic | 7679 | 1443 | 66 | 639 | 131 | 6 | 0.95 (0.79 to 1.15) | 5.94E-01 |
| rs9383938 | Intergenic | 7563 | 1568 | 104 | 644 | 133 | 9 | 1.00 (0.84 to 1.20) | 9.98E-01 |
| rs2046210 | Intergenic | 3207 | 3633 | 1069 | 304 | 370 | 93 | 1.00 (0.90 to 1.12) | 9.38E-01 |
| rs13281615 | Intergenic | 2544 | 3773 | 1455 | 264 | 366 | 133 | 1.07 (0.96 to 1.20) | 1.96E-01 |
| rs1562430 | Intergenic | 3392 | 4347 | 1496 | 262 | 384 | 140 | 0.92 (0.83 to 1.02) | 1.26E-01 |
| rs1011970 | CDKN2BAS | 6327 | 2623 | 258 | 504 | 245 | 31 | 0.85 (0.74 to 0.98) | 2.06E-02 |
| rs865686 | Intergenic | 3847 | 4247 | 1125 | 301 | 383 | 100 | 0.92 (0.83 to 1.03) | 1.61E-01 |
| rs2380205 | Intergenic | 2961 | 4505 | 1742 | 275 | 363 | 150 | 1.04 (0.94 to 1.16) | 4.19E-01 |
| rs10995190 | ZNF365 | 6818 | 2172 | 172 | 592 | 185 | 11 | 1.02 (0.87 to 1.19) | 8.43E-01 |
| rs16917302 | ZNF365 | 7599 | 1574 | 86 | 639 | 141 | 5 | 1.00 (0.84 to 1.20) | 9.89E-01 |
| rs1250003^g^ | ZMIZ1 | 3395 | 4394 | 1432 | 278 | 367 | 140 | 0.93 (0.84 to 1.04) | 2.24E-01 |
| rs3750817 | FGFR2 | 3146 | 3615 | 1063 | 313 | 358 | 101 | 1.02 (0.92 to 1.14) | 6.75E-01 |
| rs2981582 | FGFR2 | 2469 | 3868 | 1546 | 241 | 394 | 137 | 1.05 (0.94 to 1.16) | 4.21E-01 |
| rs3817198 | LSP1 | 3657 | 3387 | 821 | 339 | 351 | 80 | 0.97 (0.86 to 1.09) | 5.87E-01 |
| rs909116 | LSP1 | 2610 | 4586 | 2040 | 230 | 382 | 171 | 1.03 (0.92 to 1.14) | 6.06E-01 |
| rs614367 | Intergenic | 5119 | 1937 | 226 | 310 | 105 | 10 | 1.16 (0.95 to 1.41) | 1.43E-01 |
| rs999737^h^ | RAD51L1 | 4829 | 2702 | 401 | 483 | 245 | 41 | 1.06 (0.93 to 1.21) | 3.97E-01 |
| rs3803662 | TNRC9 | 3655 | 3328 | 797 | 357 | 324 | 76 | 0.99 (0.88 to 1.11) | 8.71E-01 |
| rs2075555 | COL1A1 | 5851 | 1856 | 165 | 580 | 177 | 9 | 1.11 (0.94 to 1.30) | 2.14E-01 |
| rs6504950 | COX11 | 4296 | 3104 | 547 | 412 | 308 | 53 | 0.98 (0.87 to 1.11) | 7.97E-01 |
| rs12982178 | USHBP1 | 6028 | 2990 | 327 | 484 | 264 | 37 | 0.89 (0.78 to 1.02) | 8.42E-02 |
| rs8170 | C19Orf62 | 6237 | 2816 | 290 | 503 | 247 | 33 | 0.90 (0.78 to 1.03) | 1.17E-01 |
| rs2284378^i^ | RALY | 4080 | 3624 | 899 | 312 | 282 | 70 | 1.01 (0.89 to 1.14) | 9.22E-01 |
| rs4911414 | Intergenic | 4177 | 3954 | 1048 | 347 | 347 | 90 | 1.01 (0.90 to 1.12) | 9.11E-01 |
| rs311499^j^ | GMEB2 | 7987 | 1162 | 66 | 664 | 110 | 7 | 0.88 (0.73 to 1.08) | 2.18E-01 |

| ^a^ The first allele is the major, the second is the minor allele  ^b^ M= Major allele; m= minor allele  ^c^ 5p12-rs4415084 or surrogate 5p12-rs920329  ^d^ 6q14-rs1917063 or surrogate 6q14-rs9344208  ^e^ 6q14-rs9344191 or surrogate 6q14-rs9449341 | ^f^ *ECHDC1R, NF146*-rs2180341 or surrogate *ECHDC1R, NF146*-rs9398840  ^g^ ZMIZ1-rs1250003 or surrogate ZMIZ1-rs704010  ^h^ *RAD51L1*-rs999737 or surrogate *RAD51L1-*rs10483813  ^i^ *RALY*-rs2284378 or surrogate *RALY*-rs6059651, *RALY*-rs8119937  ^j^ *GMEB2*-rs311499 or surrogate *GMEB2-*rs311498 |
| --- | --- |
