# Supplementary material for: Genetic risk variants associated with in situ breast cancer
Source: Breast Cancer Res. 2015 Jun 13;17(1):82. doi: 10.1186/s13058-015-0596-x (PMC4487950; doi:10.1186/s13058-015-0596-x)
Supplement: Additional file 4: — Subgroup analyses, risk of ductal breast cancer in situ (DCIS) and invasive breast cancer using distinct matched controls. [file 13058_2015_596_MOESM4_ESM.doc]

**Additional file 4. Sub-group analyses, risk of ductal breast cancer in situ (DCIS) and invasive breast cancer using distinct matched controls**

| **SNP** | **Gene or region** | **Alleles** | | **Stratum** | **Cases** | | | **Controls** | | | **OR (95% CI)** | **Ptrend** |
| --- | --- | --- | --- | --- | --- | --- | --- | --- | --- | --- | --- | --- |
|  |  |  |  |  | **MM** | **Mm** | **mm^b^** | **MM** | **Mm** | **mm^b^** |  |  |
| rs11249433 | NOTCH2 | T | G | ALL | 2659 | 4000 | 1530 | 3730 | 5044 | 1764 | 1.11 (1.06 to 1.15) | 2.07E-06 |
|  |  |  |  | DCIS | 256 | 372 | 140 | 746 | 1017 | 380 | 1.05 (0.93 to 1.18) | 4.53E-01 |
|  |  |  |  | invasive | 2403 | 3628 | 1390 | 2984 | 4027 | 1384 | 1.12 (1.07 to 1.18) | 7.90E-07 |
| rs10931936 | CASP8 | G | T | ALL | 4586 | 3815 | 772 | 5442 | 4282 | 791 | 1.07 (1.02 to 1.11) | 4.40E-03 |
|  |  |  |  | DCIS | 371 | 316 | 54 | 1084 | 831 | 166 | 1.05 (0.91 to 1.20) | 5.14E-01 |
|  |  |  |  | invasive | 4215 | 3499 | 718 | 4358 | 3451 | 625 | 1.07 (1.02 to 1.12) | 6.61E-03 |
| rs1045485 | CASP8 | G | G | ALL | 4494 | 1271 | 103 | 5296 | 1640 | 123 | 0.94 (0.87 to 1.01) | 7.28E-02 |
|  |  |  |  | DCIS | 321 | 80 | 11 | 1060 | 330 | 33 | 0.87 (0.69 to 1.10) | 2.56E-01 |
|  |  |  |  | invasive | 4173 | 1191 | 92 | 4236 | 1310 | 90 | 0.95 (0.87 to 1.03) | 2.08E-01 |
| rs13387042 | Intergenic | A | G | ALL | 2566 | 3815 | 1755 | 2791 | 5288 | 2526 | 0.86 (0.83 to 0.90) | 9.20E-13 |
|  |  |  |  | DCIS | 232 | 366 | 170 | 538 | 1088 | 513 | 0.89 (0.79 to 1.00) | 4.72E-02 |
|  |  |  |  | invasive | 2334 | 3449 | 1585 | 2253 | 4200 | 2013 | 0.87 (0.83 to 0.91) | 1.14E-09 |
| rs4973768 | SLC4A7 | G | T | ALL | 2040 | 4119 | 2020 | 2827 | 5289 | 2471 | 1.07 (1.02 to 1.11) | 2.32E-03 |
|  |  |  |  | DCIS | 188 | 375 | 202 | 581 | 1043 | 523 | 1.07 (0.95 to 1.21) | 2.32E-01 |
|  |  |  |  | invasive | 1852 | 3744 | 1818 | 2246 | 4246 | 1948 | 1.07 (1.02 to 1.12) | 5.09E-03 |
| rs4415084^c^ | Intergenic | G | T | ALL | 2657 | 3981 | 1480 | 3796 | 5033 | 1751 | 1.10 (1.06 to 1.15) | 5.81E-06 |
|  |  |  |  | DCIS | 232 | 392 | 147 | 748 | 1026 | 371 | 1.14 (1.01 to 1.28) | 3.66E-02 |
|  |  |  |  | invasive | 2425 | 3589 | 1333 | 3048 | 4007 | 1380 | 1.11 (1.06 to 1.16) | 1.69E-05 |
| rs10941679 | Intergenic | A | G | ALL | 4303 | 3236 | 612 | 5940 | 3875 | 635 | 1.15 (1.10 to 1.21) | 1.87E-09 |
|  |  |  |  | DCIS | 372 | 304 | 55 | 1161 | 784 | 123 | 1.20 (1.04 to 1.38) | 1.02E-02 |
|  |  |  |  | invasive | 3931 | 2932 | 557 | 4779 | 3091 | 512 | 1.15 (1.09 to 1.21) | 7.63E-08 |
| rs10069690 | TERT | G | T | ALL | 4412 | 3148 | 569 | 5921 | 3917 | 741 | 1.05 (1.00 to 1.10) | 5.94E-02 |
|  |  |  |  | DCIS | 436 | 282 | 52 | 1123 | 810 | 164 | 0.88 (0.77 to 1.01) | 6.50E-02 |
|  |  |  |  | invasive | 3976 | 2866 | 517 | 4798 | 3107 | 577 | 1.07 (1.01 to 1.13) | 1.19E-02 |
| rs889312 | MAP3K1 | A | G | ALL | 4016 | 3386 | 746 | 5561 | 4264 | 811 | 1.12 (1.07 to 1.17) | 1.17E-06 |
|  |  |  |  | DCIS | 393 | 304 | 80 | 1123 | 869 | 158 | 1.12 (0.98 to 1.27) | 1.02E-01 |
|  |  |  |  | invasive | 3623 | 3082 | 666 | 4438 | 3395 | 653 | 1.13 (1.07 to 1.18) | 3.69E-06 |
| rs17530068 | Intergenic | T | G | ALL | 5373 | 3512 | 598 | 6325 | 3946 | 628 | 1.05 (1.01 to 1.10) | 2.63E-02 |
|  |  |  |  | DCIS | 472 | 263 | 50 | 1257 | 730 | 137 | 0.96 (0.84 to 1.11) | 6.01E-01 |
|  |  |  |  | invasive | 4901 | 3249 | 548 | 5068 | 3216 | 491 | 1.06 (1.01 to 1.12) | 2.02E-02 |
| rs13437553 | Intergenic | T | G | ALL | 3544 | 2231 | 354 | 4309 | 2571 | 397 | 1.05 (0.99 to 1.11) | 8.29E-02 |
|  |  |  |  | DCIS | 249 | 128 | 25 | 480 | 268 | 53 | 0.93 (0.76 to 1.13) | 4.58E-01 |
|  |  |  |  | invasive | 3295 | 2103 | 329 | 3829 | 2303 | 344 | 1.06 (1.00 to 1.13) | 4.89E-02 |
| rs1917063^d^ | Intergenic | G | T | ALL | 5618 | 3379 | 513 | 6605 | 3767 | 554 | 1.05 (1.00 to 1.10) | 3.76E-02 |
|  |  |  |  | DCIS | 476 | 266 | 43 | 1318 | 720 | 119 | 1.00 (0.87 to 1.15) | 9.86E-01 |
|  |  |  |  | invasive | 5142 | 3113 | 470 | 5287 | 3047 | 435 | 1.05 (1.00 to 1.11) | 4.84E-02 |
| rs9344191^e^ | Intergenic | T | G | ALL | 5155 | 3635 | 664 | 6063 | 4080 | 710 | 1.05 (1.00 to 1.10) | 3.16E-02 |
|  |  |  |  | DCIS | 444 | 276 | 57 | 1195 | 779 | 152 | 0.97 (0.85 to 1.12) | 7.08E-01 |
|  |  |  |  | invasive | 4711 | 3359 | 607 | 4868 | 3301 | 558 | 1.06 (1.01 to 1.11) | 1.85E-02 |
| rs2180341^f^ | RNF146 | A | G | ALL | 4752 | 2927 | 505 | 6068 | 3907 | 611 | 0.99 (0.94 to 1.04) | 6.31E-01 |
|  |  |  |  | DCIS | 436 | 274 | 58 | 1229 | 762 | 112 | 1.11 (0.96 to 1.27) | 1.52E-01 |
|  |  |  |  | invasive | 4316 | 2653 | 447 | 4839 | 3145 | 499 | 0.97 (0.92 to 1.02) | 2.01E-01 |
| rs3757318 | Intergenic | G | A | ALL | 7905 | 1481 | 69 | 9186 | 1561 | 50 | 1.13 (1.06 to 1.22) | 6.02E-04 |
|  |  |  |  | DCIS | 639 | 131 | 6 | 1857 | 280 | 4 | 1.37 (1.10 to 1.71) | 5.37E-03 |
|  |  |  |  | invasive | 7266 | 1350 | 63 | 7329 | 1281 | 46 | 1.10 (1.02 to 1.19) | 1.78E-02 |
| rs9383938 | Intergenic | G | T | ALL | 7800 | 1604 | 111 | 9084 | 1739 | 83 | 1.11 (1.04 to 1.19) | 2.51E-03 |
|  |  |  |  | DCIS | 644 | 133 | 9 | 1812 | 331 | 11 | 1.12 (0.91 to 1.37) | 3.02E-01 |
|  |  |  |  | invasive | 7156 | 1471 | 102 | 7272 | 1408 | 72 | 1.10 (1.02 to 1.19) | 1.01E-02 |
| rs2046210 | Intergenic | G | T | ALL | 3309 | 3762 | 1092 | 4544 | 4813 | 1269 | 1.09 (1.04 to 1.13) | 1.75E-04 |
|  |  |  |  | DCIS | 304 | 370 | 93 | 922 | 962 | 261 | 1.05 (0.93 to 1.19) | 4.55E-01 |
|  |  |  |  | invasive | 3005 | 3392 | 999 | 3622 | 3851 | 1008 | 1.09 (1.04 to 1.14) | 3.80E-04 |
| rs13281615 | Intergenic | A | G | ALL | 2637 | 3894 | 1488 | 3618 | 5034 | 1831 | 1.06 (1.02 to 1.11) | 5.97E-03 |
|  |  |  |  | DCIS | 264 | 366 | 133 | 722 | 1048 | 345 | 1.02 (0.90 to 1.15) | 7.87E-01 |
|  |  |  |  | invasive | 2373 | 3528 | 1355 | 2896 | 3986 | 1486 | 1.06 (1.01 to 1.11) | 2.15E-02 |
| rs1562430 | Intergenic | T | G | ALL | 3477 | 4481 | 1558 | 3644 | 5330 | 1939 | 0.91 (0.87 to 0.95) | 3.22E-06 |
|  |  |  |  | DCIS | 262 | 384 | 140 | 721 | 1058 | 374 | 1.03 (0.91 to 1.16) | 6.75E-01 |
|  |  |  |  | invasive | 3215 | 4097 | 1418 | 2923 | 4272 | 1565 | 0.90 (0.86 to 0.94) | 4.58E-06 |
| rs1011970 | CDKN2BAS | G | T | ALL | 6462 | 2741 | 278 | 7616 | 2955 | 295 | 1.09 (1.03 to 1.14) | 1.92E-03 |
|  |  |  |  | DCIS | 504 | 245 | 31 | 1536 | 555 | 61 | 1.31 (1.12 to 1.52) | 6.78E-04 |
|  |  |  |  | invasive | 5958 | 2496 | 247 | 6080 | 2400 | 234 | 1.06 (1.00 to 1.13) | 4.22E-02 |
| rs865686 | Intergenic | T | G | ALL | 3950 | 4397 | 1149 | 4310 | 5004 | 1602 | 0.90 (0.87 to 0.94) | 1.09E-06 |
|  |  |  |  | DCIS | 301 | 383 | 100 | 831 | 1005 | 320 | 0.97 (0.86 to 1.10) | 6.06E-01 |
|  |  |  |  | invasive | 3649 | 4014 | 1049 | 3479 | 3999 | 1282 | 0.89 (0.85 to 0.93) | 6.79E-07 |
| rs2380205 | Intergenic | G | T | ALL | 3080 | 4628 | 1794 | 3335 | 5370 | 2187 | 0.94 (0.90 to 0.98) | 1.79E-03 |
|  |  |  |  | DCIS | 275 | 363 | 150 | 688 | 1053 | 411 | 0.95 (0.84 to 1.07) | 3.55E-01 |
|  |  |  |  | invasive | 2805 | 4265 | 1644 | 2647 | 4317 | 1776 | 0.93 (0.89 to 0.97) | 6.09E-04 |
| rs10995190 | ZNF365 | G | A | ALL | 7043 | 2227 | 173 | 7851 | 2780 | 225 | 0.89 (0.84 to 0.94) | 6.75E-05 |
|  |  |  |  | DCIS | 592 | 185 | 11 | 1521 | 589 | 46 | 0.82 (0.69 to 0.98) | 3.13E-02 |
|  |  |  |  | invasive | 6451 | 2042 | 162 | 6330 | 2191 | 179 | 0.90 (0.85 to 0.96) | 1.13E-03 |
| rs16917302 | ZNF365 | A | G | ALL | 7814 | 1635 | 85 | 8883 | 1945 | 100 | 0.96 (0.90 to 1.03) | 2.30E-01 |
|  |  |  |  | DCIS | 639 | 141 | 5 | 1752 | 378 | 21 | 0.97 (0.79 to 1.19) | 7.64E-01 |
|  |  |  |  | invasive | 7175 | 1494 | 80 | 7131 | 1567 | 79 | 0.96 (0.89 to 1.03) | 2.71E-01 |
| rs1250003^g^ | ZMIZ1 | A | G | ALL | 3475 | 4516 | 1506 | 4194 | 5054 | 1645 | 1.06 (1.02 to 1.10) | 5.35E-03 |
|  |  |  |  | DCIS | 278 | 367 | 140 | 816 | 1015 | 316 | 1.08 (0.95 to 1.21) | 2.27E-01 |
|  |  |  |  | invasive | 3197 | 4149 | 1366 | 3378 | 4039 | 1329 | 1.06 (1.01 to 1.10) | 1.37E-02 |
| rs3750817 | FGFR2 | G | T | ALL | 3245 | 3750 | 1104 | 3806 | 5096 | 1726 | 0.86 (0.83 to 0.90) | 6.40E-12 |
|  |  |  |  | DCIS | 313 | 358 | 101 | 768 | 1027 | 351 | 0.84 (0.74 to 0.95) | 4.65E-03 |
|  |  |  |  | invasive | 2932 | 3392 | 1003 | 3038 | 4069 | 1375 | 0.86 (0.82 to 0.90) | 8.59E-10 |
| rs2981582 | FGFR2 | G | T | ALL | 2566 | 3998 | 1585 | 3889 | 5066 | 1660 | 1.21 (1.16 to 1.26) | 1.45E-18 |
|  |  |  |  | DCIS | 241 | 394 | 137 | 802 | 1015 | 331 | 1.23 (1.09 to 1.39) | 1.05E-03 |
|  |  |  |  | invasive | 2325 | 3604 | 1448 | 3087 | 4051 | 1329 | 1.20 (1.15 to 1.26) | 5.65E-15 |
| rs3817198 | LSP1 | T | G | ALL | 3762 | 3538 | 839 | 4806 | 4656 | 1092 | 0.98 (0.94 to 1.03) | 4.58E-01 |
|  |  |  |  | DCIS | 339 | 351 | 80 | 964 | 919 | 249 | 1.02 (0.90 to 1.15) | 7.96E-01 |
|  |  |  |  | invasive | 3423 | 3187 | 759 | 3842 | 3737 | 843 | 0.98 (0.94 to 1.03) | 5.03E-01 |
| rs909116 | LSP1 | T | G | ALL | 2671 | 4726 | 2114 | 2997 | 5393 | 2508 | 0.97 (0.94 to 1.01) | 1.61E-01 |
|  |  |  |  | DCIS | 230 | 382 | 171 | 613 | 1039 | 501 | 0.92 (0.82 to 1.04) | 1.68E-01 |
|  |  |  |  | invasive | 2441 | 4344 | 1943 | 2384 | 4354 | 2007 | 0.98 (0.94 to 1.02) | 3.03E-01 |
| rs614367 | Intergenic | G | T | ALL | 5097 | 1891 | 216 | 5400 | 1778 | 167 | 1.14 (1.07 to 1.22) | 3.85E-05 |
|  |  |  |  | DCIS | 310 | 105 | 10 | 1038 | 360 | 26 | 1.05 (0.84 to 1.33) | 6.50E-01 |
|  |  |  |  | invasive | 4787 | 1786 | 206 | 4362 | 1418 | 141 | 1.15 (1.07 to 1.23) | 7.36E-05 |
| rs999737^h^ | RAD51L1 | G | T | ALL | 4985 | 2788 | 417 | 6240 | 3744 | 627 | 0.92 (0.88 to 0.97) | 1.15E-03 |
|  |  |  |  | DCIS | 483 | 245 | 41 | 1280 | 732 | 135 | 0.89 (0.77 to 1.02) | 9.32E-02 |
|  |  |  |  | invasive | 4502 | 2543 | 376 | 4960 | 3012 | 492 | 0.93 (0.88 to 0.98) | 6.42E-03 |
| rs3803662 | TNRC9 | G | T | ALL | 3790 | 3422 | 808 | 5542 | 4146 | 787 | 1.22 (1.16 to 1.27) | 2.54E-17 |
|  |  |  |  | DCIS | 357 | 324 | 76 | 1177 | 785 | 156 | 1.30 (1.14 to 1.48) | 8.77E-05 |
|  |  |  |  | invasive | 3433 | 3098 | 732 | 4365 | 3361 | 631 | 1.19 (1.13 to 1.26) | 6.42E-12 |
| rs2075555 | COL1A1 | G | A | ALL | 6048 | 1920 | 163 | 7957 | 2444 | 197 | 1.04 (0.98 to 1.11) | 1.80E-01 |
|  |  |  |  | DCIS | 580 | 177 | 9 | 1580 | 519 | 41 | 0.88 (0.73 to 1.05) | 1.66E-01 |
|  |  |  |  | invasive | 5468 | 1743 | 154 | 6377 | 1925 | 156 | 1.08 (1.01 to 1.15) | 3.04E-02 |
| rs6504950 | COX11 | G | A | ALL | 4450 | 3188 | 565 | 5595 | 4216 | 825 | 0.94 (0.90 to 0.98) | 5.94E-03 |
|  |  |  |  | DCIS | 412 | 308 | 53 | 1141 | 835 | 175 | 0.98 (0.85 to 1.12) | 7.27E-01 |
|  |  |  |  | invasive | 4038 | 2880 | 512 | 4454 | 3381 | 650 | 0.94 (0.89 to 0.99) | 1.25E-02 |
| rs12982178 | USHBP1 | T | G | ALL | 6174 | 3101 | 350 | 7120 | 3478 | 459 | 0.99 (0.95 to 1.04) | 7.98E-01 |
|  |  |  |  | DCIS | 484 | 264 | 37 | 1409 | 655 | 94 | 1.10 (0.95 to 1.28) | 1.83E-01 |
|  |  |  |  | invasive | 5690 | 2837 | 313 | 5711 | 2823 | 365 | 0.97 (0.92 to 1.03) | 3.38E-01 |
| rs8170 | C19Orf62 | G | A | ALL | 6391 | 2919 | 310 | 7353 | 3281 | 404 | 0.99 (0.94 to 1.04) | 7.88E-01 |
|  |  |  |  | DCIS | 503 | 247 | 33 | 1443 | 629 | 77 | 1.09 (0.94 to 1.27) | 2.41E-01 |
|  |  |  |  | invasive | 5888 | 2672 | 277 | 5910 | 2652 | 327 | 0.97 (0.92 to 1.03) | 3.62E-01 |
| rs2284378^i^ | RALY | G | T | ALL | 4158 | 3694 | 909 | 4696 | 4408 | 1026 | 0.99 (0.94 to 1.03) | 5.06E-01 |
|  |  |  |  | DCIS | 312 | 282 | 70 | 580 | 546 | 153 | 0.93 (0.81 to 1.08) | 3.47E-01 |
|  |  |  |  | invasive | 3846 | 3412 | 839 | 4116 | 3862 | 873 | 0.99 (0.95 to 1.04) | 7.72E-01 |
| rs4911414 | Intergenic | G | T | ALL | 4300 | 4083 | 1073 | 4835 | 4778 | 1239 | 0.98 (0.94 to 1.02) | 3.98E-01 |
|  |  |  |  | DCIS | 347 | 347 | 90 | 901 | 942 | 288 | 0.94 (0.83 to 1.07) | 3.39E-01 |
|  |  |  |  | invasive | 3953 | 3736 | 983 | 3934 | 3836 | 951 | 0.99 (0.95 to 1.04) | 7.66E-01 |
| rs311499^j^ | GMEB2 | G | T | ALL | 8211 | 1208 | 71 | 9405 | 1438 | 64 | 0.99 (0.92 to 1.07) | 7.60E-01 |
|  |  |  |  | DCIS | 664 | 110 | 7 | 1880 | 257 | 15 | 1.17 (0.94 to 1.46) | 1.63E-01 |
|  |  |  |  | invasive | 7547 | 1098 | 64 | 7525 | 1181 | 49 | 0.98 (0.90 to 1.06) | 5.68E-01 |

| ^a^ The first allele is the major, the second is the minor allele  ^b^ M= Major allele; m= minor allele  ^c^ 5p12-rs4415084 or surrogate 5p12-rs920329  ^d^ 6q14-rs1917063 or surrogate 6q14-rs9344208  ^e^ 6q14-rs9344191 or surrogate 6q14-rs9449341 | ^f^ *ECHDC1R, NF146*-rs2180341 or surrogate *ECHDC1R, NF146*-rs9398840  ^g^ ZMIZ1-rs1250003 or surrogate ZMIZ1-rs704010  ^h^ *RAD51L1*-rs999737 or surrogate *RAD51L1-*rs10483813  ^i^ *RALY*-rs2284378 or surrogate *RALY*-rs6059651, *RALY*-rs8119937  ^j^ *GMEB2*-rs311499 or surrogate *GMEB2-*rs311498 |
| --- | --- |
